# Southern Dental Association—1894

**Published:** 1894-11

**Authors:** J. M. Walker


### PROCEEDINGS.

### Southern Dental Association.—1894.

Twenty-fifth Annual Meeting, Old Point Comfort, Va., August 2-6, 1894.

Reported for the DENTAL REGISTER by Mrs. J. M. Walker.

[*Continued from October Number, page* 488.]

### HYGIENE.

The report of DR. W. E. WALKER, Pass Christian and Bay St. Louis, Miss., chairman of this section, was read by the secretary. The report reviewed briefly the literature of the journals on this subject during the past two years. He said—accepting as the definition of Hygiene—"that branch of medicine of which the object is the preservation of health," and including in dental hygiene all that pertains to the preservation of the teeth, the health of the mouth and its tissues, the health of the dentist and the hygiene of his surroundings, this subject covers a broad field, with many subdivisions. The papers which have appeared in the dental journals during the past two years may be classified as dental hygiene *per se*; the education of patients and the public in dental hygiene; the hygienic care of children's teeth, accepting another definition of hygiene, viz: "The philosophy of prevention in contradistinction to resistance and cure;" the special hygiene of the dentist and his surroundings; tobacco, from a hygienic point of view *pro* and *con*; hygienic precautions in the sterilization of instruments, the hands, etc.; hypnosis in its hygienic aspects; hygiene in the prevention or the arrest of decay, and last but not least, hygienic precautions in handling bank notes, quoting from the address of Dr. Ghion read before the Pan American Medical Congress in 1893, the alarming statement that "19,000 microbes of diphtheria, scarlet fever and tuberculosis have been found in a single bank note."

Dr. R. R. Freeman read a paper from Dr. T. C. West, Natchez, on "Oral Hygiene, especially in its connection with digestion." The mouths of many patients are rendered anything but pleasant to work upon owing to imperfect digestion. If the mouth were kept clean and the teeth kept in proper condition digestion would be better. The first stage of digestion is the conversion of food into a pulp-like mass which is accomplished by mastication and insalivation, in which the teeth, tongue and the salivary glands each have an important part to perform. Perfect mastication is not possible with imperfect organs for its performance, any more than a surgeon can perform perfect operations with broken instruments; the food, in the process of mastication and insalivation, if mixed with the debris from an unclean mouth and diseased teeth, is not in normal condition for reception by the stomach. Uncleanliness is a prime factor in nearly all diseases of the oral cavity. Patients must be taught the proper hygienic means of preventing this condition of things, though we too often fail in setting the proper example. Our first duty to our patients is to teach them how to care for the mouth and teeth. If an artificial denture is worn, they should be taught how to care for this. Whatever our work, whether fillings or crowns, plates or bridges, allow no spaces for the retention of particles of food to decompose in the mouth. Our work too often fails because we do not teach our patients how to care for it. Such teaching will be met with an appreciation that will amply repay the time and trouble bestowed upon it.

Dr. R. R. Freeman, Nashville, Tenn., read a paper entitled: "Hygiene."

As defined by Dr. Freeman, we understand by hygiene "the laws of health applied, whereby a man may come to his full vigor of life and accomplish that measure of usefulness and enjoy that happiness which human existence may afford." A knowledge of this science secures the means of battling against conditions likely to produce discomfort, pain, sickness or death. To appreciate the present relations of life and secure its greatest benefits, man must recognize his dual relations and act on the hypothesis of a spiritual or soul existence as well as a physical life; otherwise, the question "is life worth living?" might well be asked. Self-preservation is the first law of nature, but revelation has taught us to help the weak, succor the needy, and bind up the wounds of the afflicted. The violation of law entails calamitous results, whether against person, church or State. Health, happiness and prosperity are attainable only through the application of nature's laws. The Great Physician taught not only the value of the soul, but that its temple—these bodies of ours—must in no wise be neglected. The salvation of both soul and body is attained only through the good we do to our fellowmen. We, of the dental profession play no small part in helping to make it easy for our fellowmen to realize the blessings of life. Man may yet attain a great mastery through the science of hygiene. The light of truth and reason is fast dispelling the darkness; and honest, conscientious thinkers need no longer dread the opprobrious epithets of *crank* and *dreamer* because they maintain there is power and efficiency in the application of some of the agencies which have been discovered through the study of psychology, hypnology, etc., which if rightfully applied might soon hasten the reign of our lovely queen Hygeia. Why should the care of our bodies and the maintenance of health be deemed of less value than the æsthetic adornment of the dwellings we inhabit? The times are ripe for the advent of a new specialist—the Counselor of Hygiene.

Dr. Geo. J. Friedrichs, New Orleans, read a paper entitled: "Dental Caries and some Popular Fallacies." Among the causes most popularly assigned as factors in producing caries Dr. Friedrichs pronounced adversely upon such "causes of decay" as sweets, hot drinks, climate, soft foods, lack of cleanliness, insufficient use of the teeth, etc., his conclusion being: Given a perfectly organized denture, an unimpaired nervous system in a sound body, without inherited taint, and the teeth would never decay so long as this condition was maintained, irrespective of climate, food, or external agents; even cleanliness, now considered so essential, would be unnecessary, and be practiced only for comfort.

Dr. Cowardin thinks that the unhygienic diet of children, especially in the injudicious use of "sweets" to the exclusion of proper food is largely the cause of the poor teeth of so many children.

Dr. R. R. Freeman, on the other hand, believes that the appetite for "sweets" is a natural appetite in children, and that a proper indulgence in this respect makes them happy, and consequently, healthy. He quoted himself as still a child in that respect, still indulging freely in sweets, yet having a denture above the average for a man of his age. He said: Nothing touches me more than to see children deprived of their legitimate sweets! No matter how miserable or how pouting a child may be, they can't look cross while eating sugar candy. Never deprive children of what they enjoy. It is not the sweets that hurt them but the lack of other food in addition to the sweets. Candy made from pure sugar—not the cheap, chemically-adulterated stuff—makes children good natured and happy, and when they are happy they run and jump and exercise and are healthy. It is not the sweets, but it is high living, improper dressing, and enforced propriety of demeanor that is ruinous to the proper development of children and gives them the anæmic look and puny constitution.

Dr. J. Y. Crawford considers climate so conspicuous a factor in the production of dental caries that it may be classed among the zymotic diseases. Want of functional activity is also a pronounced factor. The influence of heredity he places at the tail end of the list of factors in the production of caries and irregularities. The present system of education, with the early confinement of young children to the school-room he considers conspicuously responsible for the early destruction of teeth. As only the dentist is competent to decide when the infant is fitted to leave the breast and masticate solid food, so also should he pronounce when it is prepared to masticate the food that will prepare both body and brain for the work of education. The ordinary American child, between the ages of four and nine, is not able to masticate the food that will enable it to bear the strain of the present school system. Too much money is spent at the wrong end of the line in this matter.

Before the subject of hygiene was passed Dr. A. C. Hewitt, Chicago, demonstrated from the platform his method of using chloroform as an obtundent, the patient inhaling a small amount of the vapor of chloroform from the mouth of a small vial, the effect being to deaden pain before reaching the point of anæsthesia. Dr. Hewitt claims to have used chloroform in this way, for the extraction of teeth, etc., for thirty years, and considers it absolutely safe. During that time he has never had an accident or even an alarming symptom. Being asked how he knew when he had reached the right point for the obtundent effect, Dr. Hewitt replied that he watched the countenance and noted a slight contraction of the nostrils as if there were a muscular effort to close them against the vapor; also a lazy drop of the eyelids, and an easy settling down in the chair. Inhaled in the manner described, he considered that the patient got about one part of chloroform vapor to one thousand of atmospheric air.

The Chair; There have been so many cases where the first inhalation of chloroform has proved fatal that it should not be allowed to go out as the voice of this Association that chloroform is ever absolutely safe. Although Dr. Hewitt has never had an accident, he may have been treading on the brink of the most imminent danger.

Dr. Sill, New York, thinks the methed of Dr. Hewitt possibly much safer than the ordinary mode of administering chloroform as so large a proportion of atmospheric air is inhaled. Instances were cited by several members of fatal, or nearly fatal, results from very slight inhalation of chloroform.

Dr. John S. Marshall was very emphatic in the declaration that *chloroform is dangerous.* He never administers it for the extraction of teeth unless the patient is at home, in bed, considering the recumbent position essential to safety.

Dr. Hewitt, on the contrary, believes the recumbent position a great source of danger, as the heavy vapor of chloroform settles down upon the face and clogs the lungs, while in the sitting posture, as in the dental chair, the falling vapor passes the nostrils and mouth, only the small amount being received that is voluntarily and forcibly inhaled. The question was discussed at great length by Drs. Sill, Noble, T. B. Welch, McKellops, Freeman, Friedrichs, Peirce, John S. Marshall.

Dr. John A. Daly read a paper on "Hygiene in Prosthetic

Dentistry," as reported under that section and Hygiene was passed.

The following new members were elected:

Dr. J. L. Wolf, Washington, D. C.; Dr. J. M. Quattlebaum, Columbus, N. Carolina; Dr B. B. Smith, Gainesville, Fla.; Dr. T. B. Welch, Vineland, N. J.; Dr. M. F. Finley; Washington, D. C.; Dr. Geo. Evans, New York;

### CLINICS.

The clinics constituted an important and interesting feature of the Southern Dental Association meeting.

Dr. H. E. Beach performed several surgical operations, principally extractions, one being of the first left inferior molar, being a case of necrosis of the alveolus with persistent abscess. In another case a badly-decayed left superior wisdom tooth was removed to allow the second molar, which was crowded out of position, to gravitate into place. In a third patient he removed a right superior bicuspid which was broken off under the gum, using as a local anæsthetic the following formula:

| | | |
|---|---|---|
| ℞ | Cocain - - - - | grs. viij |
| | Antipyrin - - - - | grs. xij |
| | Aqua dis - - - - | ℨ iij |

Mx

Sigma. Inject 2 to 5 minims in gum for removal of tooth.

Dr. V. H. Jackson exhibited and illustrated his "crib" appliances for regulating teeth.

Dr. S. B. Cook exhibited an appliance for regulating, made of bands and bars put together with vulcanite in which is placed a jackscrew. Dr. Cook also exhibited models at different stages of several cases which had been regulated with modifications of this appliance.

Dr. Hollingsworth gave a clinic with his original method of bridge-work.

Dr. W. G. Browne demonstrated the value of his method of using varnish for holding in position in the cavity the first large pellet of gold, into which he works strips of gold, relying on the varnish for *the start* merely of his filling, but not for securing the filling in the cavity. In this clinic Dr. Browne also demonstrated the special advantages of his new mechanical mallet.

Dr. R. R. Freeman filled the root-canals of a tooth with wax. He also made a tin and gold filling, using tin at the cervical wall, lining the buccal wall with gold to prevent discoloration and finishing the filling with thoroughly-annealed, extra-cohesive gold cylinders, making complete restoration of contour.

Dr. Geo. Evans demonstrated a new method of crowning front teeth; also a method of constructing hollow gold dummies for bridge-work.

Dr. John S. Thompson exhibited metallic models of block tin cast directly from plaster impressions, in which he makes celluloid plates.

Dr. John A. Daly described the method of making, and exhibited the method of using the Daly all-gold lining for rubber plates.

Dr. Wm. Crenshaw exhibited and used his new abscess drill, with a gauge, by means of which a blind abscess may be opened into through the gum tissue and alveolus, exactly at the point of the root of the affected tooth.

Dr. C. Sill exhibited a "traction engine" designed to draw into shape a sub-cervical band, when a superstructure of any kind is required on over-jutting crowns, without any mutilation of the tooth, avoiding the labor and pain of shaping a tooth as in ordinary methods.

Dr. W. V. B. Ames demonstrated the manipulation of a new germicidal cement, the oxy-phosphate of copper.

A number of promised clinics in crown and bridge-work, and in the working of metals, had to be abandoned, because of insufficient gas pressure.

A number of clinics on the printed program were not given, the operators not being present.

Among the clinics announced to the Association by the chairman of the Clinic Committee, Dr. J. Y. Crawford, was one on "Immediate Implantation," by Dr. W. Irving Thayer, Williamsburg, Mass. A voluntary essay by Dr. Thayer entitled "Root Filling" was also on the program.

The announcement of Dr. Thayer's clinic gave rise to a lengthy discussion of patented nostrums.

Dr. H. J. McKellops said: Dr. Thayer is, I understand, the proprietor of a secret preparation. The use of this in a clinic for our Association should not be allowed unless he proposes to demonstrate exactly what he is using.

Dr. Crawford stated that the privilege of the floor and the clinic had been extended to Dr. Thayer, as it was understood that his paper covers what he uses. He was satisfied that no unclean thing would be proffered this Association.

Dr. W. H. Morgan: The code of ethics prohibits all secret remedies. If there is no secret about his anæsthetic, it is all well and good.

Dr. Thayer said that he understood that he had given satisfaction in the matter to the chairman of both committees—clinics and voluntary essays; otherwise his clinic and paper would not have appeared in the program.

Dr. Morgan: Is the information Dr. Thayer has given free to all, or does it remain a secret with the chairman of the committees?

Dr. Thayer: I will give you all the constituents of my anæsthetic, and you can make it for yourselves, but I will not tell you how I make it.

Dr. F. Peabody: This cannot be accepted by this Association unless the proportions of the constituents, the manner of compounding, and the method of using is given fully. It is not admissible before a professional body. He must not leave us in partial ignorance of what he apparently gives us, but he must make us able to do all that he does himself.

Dr. H. B. Noble: There is nothing in Dr. Thayer's paper that is objectionable or it would not have been accepted by the committee on voluntary essays. I understood that in connection with his paper he was to give us exactly what he does and why he does it. I had no idea he intended to keep secret the way in which he prepares his preparation. If he proposes to use a secret compound and not tell us all there is about it, I withdraw my endorsement.

DR. THAYER: My paper has no reference to anything I manufacture. It has no reference to any anæsthetic I make.

DR. COWARDIN: Dr. Thayer has not stated that he will give us the complete formula of the anæsthetic he proposes to use in his clinic, with the method of preparation and application. His paper seems to be acceptable, but unless he proposes to give a complete exposition of his remedy I would suggest that he abandon it in his clinic and use something that is acceptable to the Association.

DR. PEABODY: Unless he gives it to us so that we can all do what he does, understandingly, I do not see how the profession will be benefited.

DR. R. R. FREEMAN: I understand that Dr. Thayer proposes to give us his secret, and yet having given it to you—seeing, you see not! hearing, you hear not!

DR. THAYER: I will relieve the Association from the burden of my clinic.

DR. H. E. BEACH: Having seen Dr. Thayer's paper, I can say with a clear conscience that it does not contain a single reference to anything savoring of quackery or nostrums. He simply says, "use cocaine or other anæsthetic." When Dr. Thayer proposes to give the formula of his preparation he does all that we can ask. If a competent druggist cannot compound it from his formula then he has not given the correct formula.

DR. CRAWFORD: If Dr. Thayer can give us something that will enable us to perform our operations painlessly, we want it. If he gives us his formula properly written out and so constructed that we can prescribe it, that is as far as we can go. If he is entitled to read a paper, he is entitled to clinic. If he is not entitled to clinic he cannot read a paper. He cannot be ethical in one thing and unethical in another. If you are right I am with you. If you are wrong I am against you. This is no question of legality; no question of morality. It is a question of professional ethics. If he refuses to give us what he has got he is not entitled to the confidence of his professional brothers; he is not entitled to recognition. The question is—are you in full fellowship with your profession? Have you a secret that your profession needs? Open your heart; open your purse; help us to help humanity. Not for all the money in the world would I disregard one single obligation towards my brothers. I gladly tell all the little I know. This thing of patents and secret remedies is worse than a millstone around the neck. Far from keeping anything secret, I can't get you to believe the little I do know; I know that a tooth grows, but you won't believe that! If you have got a good remedy, for God's sake give it to us; there is not money enough in the world to repay you for keeping it back. I doubt if you get to heaven if you keep it back! Give it to us for those delicate women for whom we have to cut into sensitive dentine. Give us the means of lessening the pain we inflict. Give us the formula, and give us the method by which it is prepared.

The Chair: The question is now in your hands. Shall Dr. Thayer give the proposed clinic?

Dr. Thayer: To relieve the Association of all embarrassment, both clinic and paper are withdrawn.

This discussion held the Association 'till 11 P. M!

At the last session a resolution was adopted requiring the President to appoint a Clinical Staff, an organized clinical corps, a chairman and secretary and three helpers, with a view of making the clinics more satisfactory than by past methods.

By resolution adopted by the Southern Dental Association, all charter members are exempt from further payment of dues, beginning with the twenty-fifth annual meeting 1894.

A resolution was adopted reinstating members in arrears for dues, on payment of two years' dues, $6.00. A number of old members were reinstated on these terms.

Dr. H. E. Beach, chairman of the Committee on Revision of the Constitution and By-Laws, reported that he had not been able to obtain a meeting of the committee, consequently there was no report to be presented.

On motion, the committee was continued another year. Drs. H. E. Beach, E. S. Chisholm and B. H. Catching, committee

The Treasurer, Dr. H. E. Beach, presented the following report:

Balance on hand at last report.......................$290.27
Cash paid out as per vouchers....................... 144.23
Balance on hand.........................................$146.04

Drs. W. R. Clifton and Jos. R. Woodly, the auditing committee, reported having examined the books and vouchers of the Treasurer and found everything correct.

The above statement does not include the receipts and expenditures of the present meeting, 1894.

CHAPIN A. HARRIS MEMORIAL

Dr. T. H. Parramore spoke in feeling terms of the absence of the venerable Dr. W. H. H. Thackston, who by serious illness was prevented from laying before the Association plans for the erection of a monument of suitable character to the memory of Dr. Chapin A. Harris, "the father of modern dentistry."

He asked the Southern Dental Association to take the matter in hand and to take steps to have the matter brought before the different State Associations.

After some discussion, the Corresponding Secretary, Dr. E. P. Beadles was instructed to correspond with the officers of the various State Associations with a view to raising a fund for this purpose. Dr. H. E. Beach was elected treasurer of the Chapin A. Harris Memorial fund.

Drs. B. Holly Smith, J. Y. Crawford and J. S. Thompson, were appointed a committee to lay the matter before the American Dental Association.

As a nucleus, Dr. B. H. Smith donated to the fund the sum of $240.00 raised in Baltimore four years ago, by the Baltimore Chapin A. Harris Association.

A telegram of sympathy was sent to Dr. Thackston to which the following reply was received:

"FARMVILLE, VA., August 6th, 1894.

"Loved Friends of the Southern Association:

"A thousand thanks for your kind remembrance and the sympathy expressed, which from my heart of hearts is reciprocated. "W. H. H. THACKSTON."

### THE DENTAL PROTECTIVE ASSOCIATION.

Dr. J. N. Crouse, the manager of the Association made a statement giving the present status of litigation and also the financial standing of the Association. At his request, a committee was appointed to co-operate with a similar committee for the American Dental Association to examine the books, papers, vouchers, and mortgage notes of the Dental Protective Association.

The Southern Association appointed Drs. R. R. Freeman and V. E. Turner. The American Association appointed Drs. H. A. Smith, C. E. Esterly and Louis Jack.

This joint committee reported at a session of the American Association that they had examined the books and documents submitted by Dr. Crouse, and found that the funds received were fully accounted for, and the affairs of the Association economically administered, the money on hand being invested in good mortgage securities.

Dr. Crouse stated that the case with the International Tooth Crown Co., would probably be argued in October, in Brooklyn, and that it might be necessary to make an assessment upon the members of the Association to pay the lawyer's fees, as the mortgage securities could not be realized on at present without heavy loss.

### ELECTIONS.

Atlanta, Georgia, was unanimously selected as the next place of meeting, the time being left for future announcement by the executive committee.

The election of officers resulted as follows:

Dr. H. E. Beach, Clarksville, Tenn., President.

Dr. J. S. Thompson, Atlanta, Ga., 1st Vice President.

Dr. L. P. Dotterer, Charleston, S C., 2d Vice President.

Dr. Seals, Fort Smith, Ark., 3d Vice President.

Dr. S. W. Foster, Decatur, Ala., Recording Secretary.

Dr. E. P. Beadles, Danville, Va., Corresponding Secretary.

Dr. H. A. Lowrance, Athens, Ga., Treasurer.

Dr. S. B. Cook, Chattanooga, Tenn., Dr. V. E. Turner, Raleigh, N. C., to fill vacancies on the executive committee.

The official publication of the proceedings was awarded to the Wilmington Dental Manufacturing Co., Philadelphia, the Company agreeing to publish an *edited edition* in sections in the regular issues of the *Items of Interest*, and to issue at a later date a full report in book-form for the members of the Association.

After passing the customary resolutions of thanks, etc., and the installation of the newly-elected officers, the Association adjourned to meet in Atlanta, Ga., in 1895, at a date to be announced by the Executive Committee.

